# The impact of the absolute number and ratio of positive lymph nodes on survival of endometrioid uterine cancer patients

**DOI:** 10.1038/sj.bjc.6603898

**Published:** 2007-07-31

**Authors:** J K Chan, D S Kapp, M K Cheung, K Osann, J Y Shin, D Cohn, P L Seid

**Affiliations:** 1 Division of Gynecologic Oncology, Department of Obstetrics, Gynecology, and Reproductive Sciences, University of California, San Francisco School of Medicine, San Francisco Comprehensive Cancer Center, 1600 Divisadero Street, Box 1702, San Francisco, CA 94143, USA; 2 Division of Radiation Therapy, Department of Radiation Oncology, Stanford University School of Medicine, Stanford Cancer Center, 875 Blake Wilbur Drive, MC 5827, Stanford, CA 94305, USA; 3 Department of Obstetrics and Gynecology, Division of Gynecologic Oncology, Stanford University School of Medicine, Stanford Cancer Center, 875 Blake Wilbur Drive, MC 5827, Stanford, CA 94305, USA; 4 Division of Hematology and Oncology Department of Medicine, , Chao Family Comprehensive Cancer Center, University of California, Irvine Medical Center, 101 The City Drive, Orange, CA 92868, USA; 5 Division of Gynecologic Oncology, Department of Obsetrics and Gynecology, The Ohio State University Comprehensive Cancer Center, Ohio State University College of Medicine and Public Health, M210 Starling-Loving Hall, 320 West Tenth Avenue, Columbus, OH 43210, USA

**Keywords:** positive lymph nodes, uterine cancer, prognostic factors

## Abstract

The aim of the study was to determine the impact of the absolute number and ratio of positive lymph nodes on the survival in node-positive endometrioid uterine cancer. Data were obtained from the National Cancer Institute Registry from 1988 to 2001. Analyses were performed using Kaplan–Meier and Cox proportional hazard methods. A total of 1222 women were diagnosed with stage IIIC-IV node-positive endometrioid corpus cancer. The 5-year disease-specific survival of women with 1, 2–5, and >5 positive nodes were 68.1, 55.1, and 46.1%, respectively (*P*<0.001). Increasing lymph node ratio, expressed as a percentage of positive nodes to total nodes identified (⩽10, >10–⩽50, and >50%), was associated with a decrease in survival from 77.3 to 60.7 to 40.9%, respectively (*P*<0.001). The absolute number of positive nodes and the lymph node ratio remained significant after adjusting for stage (IIIC *vs* IV) and the extent of lymphadenectomy (⩽20 *vs* >20 nodes). On multivariate analysis, the absolute number of positive nodes and lymph node ratio were significant independent prognostic factors for survival. Increasing absolute number of positive nodes and lymph node ratio are associated with a poorer survival in women with node-positive uterine cancers. The stratification of node-positive uterine cancer for prognostic and treatment purposes warrants further investigation.

Endometrial carcinoma is the most common gynaecological malignancy in the United States with an expected 7350 deaths associated with this disease in 2006 (Jemal *et al*, 2006). Although approximately 80% of patients are diagnosed with early-stage (I–II) disease and have an excellent prognosis, women with advanced-stage (III–IV) cancers have significantly poorer outcome. Metastatic involvement of retroperitoneal lymph nodes is one of the most important prognostic factors (Morrow *et al*, 1991). Of the patients with node-positive disease, the estimated 5-year disease-specific survival ranges from 10 to 75% (Potish *et al*, 1985; Larson *et al*, 1987; Lurain *et al*, 1991; Morrow *et al*, 1991; Rose *et al*, 1992; Greven *et al*, 1993; Schorge *et al*, 1996; Nelson *et al*, 1999; Mariani *et al*, 2002; Watari *et al*, 2005; Chan *et al*, 2006; Randall *et al*, 2006). The wide range of outcomes suggests that there exists considerable heterogeneity in these node-positive patients based on various clinicopathological prognostic factors. Furthermore, over 50% of stage III–IV disease patients fail standard treatments with either whole abdominal radiation or chemotherapy and experience significant toxicities (Bruner *et al*, 2006; Randall *et al*, 2006). Studies focused on defining these prognostic factors may permit better substage stratification and determine novel treatment strategies. For example, multi-modality therapies should be designed for high-risk patients to improve survival and individualised tailored therapies are preferred in low-risk patients to prevent toxicities associated with over-treatment.

The association between the extent of lymph node involvement and survival has been demonstrated in most solid tumours including lung, breast, colon, rectal, bladder, cervical, and vulva cancers (van der Velden *et al*, 1995; Moore and Stehman, 1996; Tepper *et al*, 2001; Herr *et al*, 2002; Weir *et al*, 2002; Gajra *et al*, 2003; Joseph *et al*, 2003; Le Voyer *et al*, 2003). Furthermore, the number of nodal metastases in breast cancer is not only used as a prognostic tool but it also guides adjuvant treatment (NCCN, 2006). However, the current staging system for uterine cancer under International Federation of Gynecology and Obstetrics (FIGO) does not account for the extent of nodal disease. Prior studies have shown that patients with stage IIIC uterine cancer limited only to the pelvic nodes have significantly better prognosis than for other subgroups of stage IIIC disease, suggesting that stratification may be appropriate (Morrow *et al*, 1991; Nelson *et al*, 1999; Mariani *et al*, 2002; Watari *et al*, 2005).

In this present study, we determined the prognostic significance of the absolute number and ratio of positive lymph nodes in endometrioid uterine cancer. Furthermore, we identified other clinicopathological prognostic factors important in node-positive corpus cancer.

## PATIENTS AND METHODS

Demographic, clinicopathological, treatment, and survival information of women diagnosed with endometrioid corpus cancer during the period from 1 January 1988 to 31 December 2001 was extracted with permission from the Surveillance, Epidemiology and End Results (SEER) programme of the United States National Cancer Institute (Surveillance, 2005). This data represent approximately 14% of the US population and is reported from 12 population-based registries including San Francisco-Oakland, Connecticut, metropolitan Detroit, Hawaii, Iowa, New Mexico, Seattle (Puget Sound), Utah, metropolitan Atlanta, Alaska, San Jose-Monterey, and Los Angeles.

Of the 40 880 women diagnosed with endometrioid uterine cancer, 1222 patients had stage IIIC–IV disease with at least one positive pelvic and/or paraaortic lymph node. All patients underwent surgical staging including lymphadenectomy. Information regarding patient age, stage, tumour grade, number of positive lymph nodes, extent of lymph node dissection (defined as the total number of lymph nodes recovered), and use of adjuvant radiation therapy was extracted. Patients were divided into three nodal groups (1, 2–5, and >5 positive nodes). The lymph node ratio, expressed as the percentage of positive nodes to total nodes identified, was stratified into three groups (⩽10, >10–⩽50, and >50%).

Statistical analysis was performed using the Intercooled STATA 8.0 program (College Station, TX). Survival analysis was performed using the Kaplan–Meier method. The outcome of interest was death from endometrial cancer. Time to death was censored in women who died from causes other than uterine cancer. The Cox proportional hazards model was used to identify independent predictors of survival. A forward stepwise model was used to determine which prognostic variable was more important for prediction of outcome. Two-tailed tests at *P*-values less than 0.05 were considered significant.

## RESULTS

Patient characteristics of 1222 women diagnosed with stage IIIC–IV node-positive endometrioid uterine cancer are shown in Table 1. The median age at diagnosis was 64 years (range: 28–93 years). 639 (52.3%) were stage IIIC, 24 (2.0%) were stage IVA, and 559 (45.7%) were stage IVB. The median number of lymph nodes removed was 11 (range: 1–90). The median number of positive lymph nodes was 2 (range: 1–52) and the median lymph node ratio was 23.2% (range: 1.8–100%). The 5-year disease-specific survivals of patients with stage IIIC and IV were 70.3 and 47.8%, respectively (*P*<0.001). A more extensive lymphadenectomy (*P*<0.001), lower grade tumours (*P*<0.001), and use of radiotherapy (*P*<0.001) were associated with an improved survival (Table 2).

**Figure 1:**
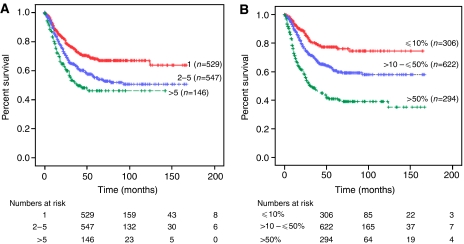
Kaplan–Meier disease-specific survival of node-positive endometrioid uterine cancer based on (**A**) absolute number (1, 2–5, and >5) of positive nodes: 68.1, 55.1, and 46.1%; *P*<0.001, and (**B**) ratio of positive lymph nodes (⩽10, >10–⩽50, and >50%): 77.3, 60.7, and 40.9%; *P*<0.001.

**Figure 2:**
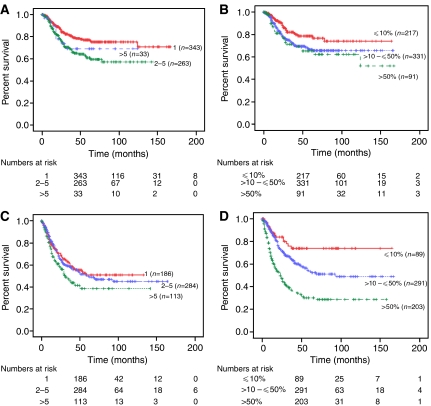
Kaplan–Meier disease-specific survival based of stage IIIC (**A** and **B**) *vs* stage IV (**C** and **D**) disease by absolute number (**A** and **C**) and ratio (**B** and **D**) of positive lymph nodes.

**Figure 3:**
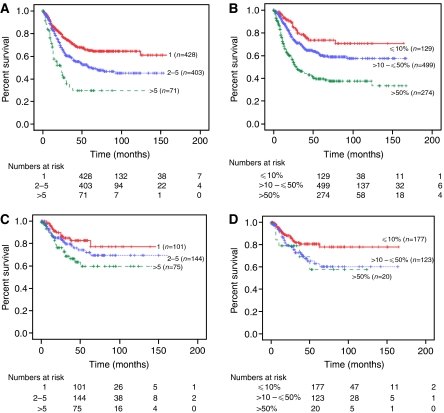
Kaplan–Meier disease-specific survival based on extent of node dissection ⩽20 nodes (**A** and **B**) *vs* >20 nodes (**C** and **D**) disease by absolute number (**A** and **C**) and ratio (**B** and **D**) of positive lymph nodes.

When all patients were divided into three groups based on the number of positive lymph nodes (1, 2–5, and >5 positive nodes), the 5-year disease-specific survival decreased from 68.1 to 55.1 to 46.1%, respectively (*P*<0.001; Figure 1). Adjusted for stage of disease, the association between absolute number (1, 2–5, and >5) of positive nodes and survival rates were 77.1, 60.9, and 69.1%, respectively (*P*=0.003) in patients with stage IIIC disease and 50.9, 49.8, and 38.9%, respectively (*P*=0.099) in stage IV cancers (Figure 2A and C). The prognostic significance of the absolute number of positive nodes was also examined based on the extent of lymphadenectomy. In those with ⩽20 nodes identified, the absolute number (1, 2–5, and >5) of positive nodes was associated with a decrease in survival from 65.1 to 50 to 29.7%, respectively (*P*<0.001). In addition, women with >20 nodes identified had an associated decrease in survival from 82.6 to 71.9 to 59.7%, respectively (*P*=0.047; Figure 3A and C) for the three positive nodal groups.

Increasing lymph node ratio (⩽10, >10–⩽50, and >50%) was associated with a decrease in 5-year disease-specific survival from 77.3 to 60.7 to 40.9%, respectively (*P*<0.001; Figure 1). For patients with stage IIIC disease, survival decreased from 78.6 to 66.5 to 65.3%, corresponding to the three lymph node ratio groups (*P*=0.025), and this finding was consistent in stage IV cancers with associated survival rates of 73.8 to 53.3 to 30.1%, respectively (*P*<0.001; Table 2 and Figure 2B and D). We also divided the study group based on the extent of lymphadenectomy and found that the group with ⩽20 nodes resected had associated survivals of 73.5, 60.1, and 39.5%, for the three corresponding lymph node ratio groups, respectively (*P*<0.001). Similarly, those with >20 nodes resected had survival rates of 80.6, 62.9, and 57.6% (*P*=0.052; Figure 3B and D). The impact of the absolute number and ratio of positive nodes by stage, grade of disease, radiation therapy, and extent of lymphadenectomy on survival is summarised in Table 2.

On multivariate analysis, the absolute number of positive lymph nodes (*P*=0.005) and the lymph node ratio (*P*=0.003) were independent prognostic factors for survival adjusting for age, stage, grade of disease, year of diagnosis, adjuvant radiation, and extent of lymphadenectomy (Table 3). Hazard ratios were higher for patients with more than 5 positive nodes relative to those with 2–5 positive or 1 positive node (HR=1.63 *vs* 1.28 *vs* 1.0, respectively). Moreover, the hazard ratio was significantly higher for each percent increase in lymph node ratio (HR=1.05 for a 10% increase in lymph node ratio). Correlation between these variables was relatively small (*r*=0.2), thus each contributed independently to the prediction of the hazard rate. In forward stepwise regression, lymph node ratio entered the model before absolute number of positive nodes suggesting an overall greater prognostic ability. However, when the analysis is restricted to patients with less extensive lymphadenectomy (⩽10 nodes), only the absolute number of positive nodes contributed significantly to prediction of survival (*P*=0.005 for absolute number of positive nodes; *P*=0.186 for lymph node ratio).

## DISCUSSION

Advanced stage uterine cancer continues to be a significant cause of death among gynaecological cancers in the United States (Jemal *et al*, 2006). Prior studies have found that stage IIIC node-positive endometrial cancers comprise of only 2–6% of all cases (Creasman *et al*, 1987; Lurain *et al*, 1991; Morrow *et al*, 1991; Greven *et al*, 1993; Schorge *et al*, 1996; Faught *et al*, 1998). Given that it is difficult for institutions to collect a large series of women with node-positive uterine cancer, this subgroup of patients has not been well studied. In addition, the prognostic factors in these high-risk patients have not been extensively characterised. In this current series of 1222 women with node-positive endometrioid uterine cancer, we determined the prognostic significance of the absolute number and ratio of positive nodes. In addition, this is one of the first papers that compared the prognostic significance of lymph node ratio to absolute number of positive nodes in patients with endometrial cancer.

Since women with node-positive endometrioid uterine cancer have a wide range of survival from 10 to 75% (Potish *et al*, 1985; Larson *et al*, 1987; Lurain *et al*, 1991; Morrow *et al*, 1991; Rose *et al*, 1992; Greven *et al*, 1993; Schorge *et al*, 1996; Faught *et al*, 1998; Nelson *et al*, 1999; Mariani *et al*, 2002; Watari *et al*, 2005; Chan *et al*, 2006; Randall *et al*, 2006), several investigators have suggested substaging these patients (Nelson *et al*, 1999; Mariani *et al*, 2002; Watari *et al*, 2005). In this current report, our findings support the concept of stratifying node-positive cancers into substages based on nodal burden. The 5-year disease-specific survival of this heterogeneous cohort of node-positive patients in our study ranges from approximately 40 to 77%.

The prognostic significance of the absolute number and/or ratio of positive nodes has been reported in other solid tumours (van der Velden *et al*, 1995; Moore and Stehman, 1996; Tepper *et al*, 2001; Bando *et al*, 2002; Herr *et al*, 2002; Weir *et al*, 2002; Gajra *et al*, 2003; Joseph *et al*, 2003; Le Voyer *et al*, 2003; Berger *et al*, 2005; Sierzega *et al*, 2006). In breast cancer, the nodal ratio has been shown to be an important predictor of loco-regional recurrence and survival both from initial diagnosis and following recurrence (Nieto *et al*, 2002; Grills *et al*, 2003; Woodward *et al*, 2006). Several studies compared the prognostic value of nodal ratios to the absolute number of positive nodes and found that lymph node ratio had a stronger prognostic value in breast cancer (Vinh-Hung *et al*, 2004; Woodward *et al*, 2006).

Small retrospective studies from single institutions have evaluated the significance of the absolute number and ratio of positive nodes in uterine cancers. Mariani *et al* (2001) studied 60 patients with endometrial cancer with pelvic nodal metastases. These authors found that patients who recurred or died of disease had a higher percentage of positive lymph nodes at presentation. Similarly, Tang *et al*, (1998) evaluated 40 patients with pelvic and/or paraaortic nodal metastases and found a 5-year disease-specific survival of 0 *vs* 55% in those with ⩾25 *vs* <25% lymph node ratio. Yasunaga *et al*, (2003) have also shown that a high metastatic ratio is associated with a lower survival. Patients with lymph node ratios of <10, 10–50, and >50% had 5-year survival rates of 82.5, 43.8, and 0%, respectively (Yasunaga *et al*, 2003). However, these studies were limited by the small sample sizes and inclusion of high-risk cell types such as serous and clear cell cancers. More importantly, there may exist a potential selection bias due to reporting from tertiary care academic centres caring for high-risk patients. Thus, these small study cohorts may not be representative of the general population. Furthermore, these studies did not compare the prognostic importance of nodal ratio to the absolute number of positive nodes, likely due to the limitations in sample size.

On multivariate analysis of the 1222 node-positive patients in this current study, the absolute number (*P*=0.005) and lymph node ratio (*P*=0.003) remained as independent prognostic factors after adjusting for age, stage, grade of disease, and extent of lymph node dissection. The correlation between the categorical variables, absolute number and ratio of positive nodes, was relatively small and thus both variables were independent predictors for survival. Analysed as a continuous variable, lymph node ratio entered the proportional hazard model before absolute number of positive nodes in stepwise regression suggesting that the lymph node ratio may better characterise prognostic subgroups than the number of positive nodes. In addition, Kaplan–Meier analyses provide further support showing a larger separation of curves depicted in the overall study group (Figure 1) and substages of IIIC *vs* IV cancers (Figure 2).

Compared to the absolute number of positive lymph nodes, the lymph node ratio may be a better predictor of tumour burden and aggressive biological behaviour of the tumour, particularly in those who had a more extensive lymphadenectomy. As such, the survival differences within these subgroups by lymph node ratio were more pronounced. However, in patients with a limited number of lymph nodes recovered, the absolute number of positive nodes appears to be a better predictor. It is important to note that the number of nodes recovered may reflect a more extensive dissection, comprehensiveness of pathological evaluation, variations in number of nodes of each patient, and difficulties in performing lymphadenectomies due to medical comorbidities (Yasunaga *et al*, 2003).

The majority of stage III–IV uterine cancer patients fail standard chemotherapy or whole abdominal radiation (Randall *et al*, 2006). It would be important to identify the patients at particularly high risk of recurrence in whom a combination of chemotherapy and radiation therapy may be beneficial, while sparing lower-risk patients from the toxicity of excessive therapy (Bruner *et al*, 2006). Extrapolating from breast cancer treatment recommendations, it may be possible that tailored therapy can be selected based on stratifying patients with node-positive uterine cancer into various risk groups. For example, local irradiation may be adequate for those with low numbers of lymph nodes involved, particularly if a thorough lymphadenectomy was performed. In contrast, higher-risk patients with a larger nodal tumour burden may warrant more extensive treatment including systemic chemotherapy combined with site-specific irradiation. In fact, a current Gynecologic Oncologic Group trial is investigating the role of multi-modality therapy in advanced uterine cancer to define the optimal chemotherapy combined with radiation (Homesley *et al*, 2000).

The finding that the overall number of lymph nodes removed at surgery correlates with survival has been previously reported (Kilgore *et al*, 1995; Blythe *et al*, 1997; McMeekin *et al*, 2001; Chan *et al*, 2006; Lutman *et al*, 2006; Mariani *et al*, 2006). In early-stage disease, it is not clear whether this benefit is due directly to cytoreductive effects of surgery or from more accurate staging. In this current study of node-positive patients, we found that a more extended dissection remained as an important predictor for survival after controlling for the absolute number and ratio of positive nodes. The improvement in survival associated with extent of lymph node resection and possible mechanisms have been previously reported (Chan *et al*, 2006). Lastly, it is possible that lymph node dissection may be a surrogate for the quality comprehensive care rather than the cause that resulted in the better outcome of these patients.

Our study has several recognisable limitations. There is a lack of information on surgeon sub-specialty, comprehensive surgical staging, central pathology review, adjuvant hormonal and chemotherapy, time to recurrence, subsequent surgical and medical therapies, and surgical morbidity. Additionally, there was limited data on the specific laterality and location of nodal resection. Moreover, our data do not have detailed information regarding the depth of myometrial invasion, peritoneal cytology, extent of nodal debulking, and extent of extrauterine involvement associated with the node-positive cancer. It is conceivable that the extent of extrauterine involvement may be strongly correlated with the lymph node burden and thus confound our findings.

The strengths of this study include the fact that this is one of the largest studies evaluating the impact of nodal burden and lymphadenectomy on the survival of node-positive endometrioid uterine cancer. In addition, all patients with high-risk cell types such as papillary serous, clear cell, and sarcomas were excluded from the analyses. The wide geographical distribution of patients including 12 US regions also decreases the potential selection and surveillance biases that are associated with single-institution analyses (Surveillance, 2005). Furthermore, the results from this population-based study can be generalised to the entire US population. The quality control measures of the SEER programme allow the registry to maintain the highest level of certification of data quality and completeness reported by the Northern American Association of Central Cancer Registries.

In summary, improving risk assessments in advanced endometrial cancers beyond the current FIGO staging definition is of particular interest as the treatment of high-risk uterine cancers evolves. The sub-classification of node-positive cancers based on the absolute number and ratio of positive nodes may assist the physician to better define prognosis and more importantly, stratify patients into various risk groups in the design of future clinical trials. If confirmed in a prospective clinical trial, these findings may ultimately modify the current staging system and lead to individualised tailored therapies in patients with node-positive uterine cancer.

## Figures and Tables

**Table 1:** Clinicopathological Characteristics of Patients with Node-Positive Endometrioid Uterine Cancer (*n*=1222)

**Parameters**	***n* (%)**
*Age (years)*	
<65	626 (51.2%)
⩾65	596 (48.8%)
	
*Race*	
White	1025 (83.9%)
Black	75 (6.1%)
Asian	91 (7.4%)
Other	31 (2.5%)
	
*Year of diagnosis*	
1988–1992	230 (18.8%)
1993–1997	455 (37.2%)
1998–2001	537 (43.9%)
	
*Stage*	
Stage IIIC	639 (52.3%)
Stage IV	583 (47.7%)
Stage IVA	24 (2.0%)
Stage IVB	559 (45.7%)
	
*Grade*	
Grade 1	123 (10.0%)
Grade 2	466 (38.1%)
Grade 3	581 (47.5%)
Unknown	52 (4.3%)
	
*Radiation*	
No XRT	423 (34.6%)
Adjuvant XRT	772 (63.2%)
Unknown	27 (2.2%)
	
*Number nodes removed*	
⩽10	582 (47.6%)
11–20	320 (26.2%)
>20	320 (26.2%)
	
*Number of metastatic nodes*	
1 positive node	529 (43.3%)
2–5 positive nodes	547 (44.8%)
>5 positive nodes	146 (11.9%)
	
*Lymph node ratio* (%)	
⩽10	306 (25.0%)
>10–⩽50	622 (50.9%)
>50	294 (24.1%)

Abbreviation: XRT, radiotherapy.

**Table 2:** Five-Year Disease-Specific Survival by Absolute Number of Positive Nodes and Ratio of Positive to Examined Nodes

		**Absolute number of positive nodes**				**Ratio of positive to examined nodes**			
	**Survival (*n*=1222) % (s.e.)**	**1 (*n*=529) % (s.e.)**	**2–5 (*n*=547) % (s.e.)**	**>5 (*n*=146) % (s.e.)**	**Log-rank *P*-value**	**⩽10% (*n*=306) % (s.e.)**	**>10–⩽50% (*n*=622) % (s.e.)**	**>50% (*n*=294) % (s.e.)**	**Log-rank *P*-value**
*Stage*									
Stage IIIC	70.3 (±2.3)	77.1 (±2.8)	60.9 (±4.0)	69.1 (±9.2)	=0.003	78.6 (±3.6)	66.5 (±3.3)	65.3 (±6.0)	=0.025
Stage IV	47.8 (±2.7)	50.9 (±4.8)	49.8 (±3.8)	38.9 (±6.1)	=0.099	73.8 (±5.7)	53.3 (±4.0)	30.1 (±4.0)	<0.001
									
*Number of nodes removed*									
⩽20	55.6 (±2.1)	65.1 (±2.9)	50.0 (±3.2)	29.7 (±6.9)	<0.001	73.5 (±4.9)	60.1 (±2.8)	39.5 (±3.6)	<0.001
>20	71.9 (±3.3)	82.6 (±4.6)	71.9 (±5.2)	59.7 (±7.1)	=0.047	80.6 (±3.7)	62.9 (±5.8)	57.6 (±14.7)	=0.052
									
*Grade*									
Grade 1	88.1 (±3.6)	92.9 (±4.0)	80.8 (±6.7)	100.0 (±0.0)	=0.10	96.7 (±3.3)	82.6 (±5.7)	93.8 (±6.1)	=0.133
Grade 2	64.6 (±2.9)	72.8 (±3.8)	57.6 (±4.7)	54.3 (±10.4)	=0.037	83.7 (±4.0)	60.8 (±4.3)	49.1 (±6.3)	<0.001
Grade 3	49.0 (±2.6)	56.4 (±4.2)	46.8 (±3.9)	39.5 (±6.1)	=0.010	65.5 (±5.3)	54.6 (±3.7)	26.7 (±4.3)	<0.001
									
*Radiation*									
No XRT	46.2 (±3.2)	49.9 (±5.4)	47.9 (±4.7)	34.6 (±7.5)	=0.051	76.5 (±6.6)	55.2 (±4.5)	25.5 (±4.5)	<0.001
Adjuvant XRT	67.2 (±2.1)	77.1 (±2.8)	59.6 (±3.5)	58.3 (±7.0)	<0.001	78.7 (±3.3)	63.7 (±3.1)	58.4 (±4.9)	<0.001

Abbreviations: s.e., standard error; XRT, radiotherapy.

**Table 3:** Multivariable Analysis for Important Prognosticators in Node-Positive Endometrioid Uterine Cancer (*n*=1222)

	**Hazard ratio**	**95% CI**	***P*-value**
Age at diagnosis^a^	1.03	(1.02–1.04)	<0.0005
Stage of disease^b^	1.66	(1.31–2.10)	<0.0005
Grade^c^	2.01	(1.66–2.42)	<0.0005
Radiation^d^	0.61	(0.49–0.76)	<0.0005
Year of diagnosis^e^	0.70	(0.55–0.88)	=0.002
Extent of lymphadenectomy^f^	0.60	(0.44–0.82)	=0.001
Positive lymph nodes^g^	1.28	(1.08–1.51)	=0.005
Lymph node ratio^h^	1.70	(1.20–2.40)	=0.003

a Age at diagnosis as a continuous variable.

b Stage of disease as IIIC *vs* IV.

c Grade as 1 *vs* 2 *vs* 3.

d No radiation *vs* adjuvant radiation.

e Year of diagnosis as ⩽1992 *vs* >1992.

f Extent of lymphadenectomy as 1–20 *vs* >20 nodes.

g Positive lymph nodes as 1 *vs* 2–5 *vs* >5 positive nodes.

h Lymph node ratio as a continuous variable.
